# Special Issue: The Antioxidant Capacities of Natural Products

**DOI:** 10.3390/molecules24030492

**Published:** 2019-01-30

**Authors:** Susana M. Cardoso

**Affiliations:** QOPNA & LAQV-REQUIMTE, Department of Chemistry, University of Aveiro, 3810-193 Aveiro, Portugal; susanacardoso@ua.pt

Antioxidants are under the research spotlight because of their potential to prevent oxidative stress as well as for their versatile biological properties that grant them multiple industrial applications. This Special Issue of *Molecules* collects 30 original research articles covering several aspects of the antioxidant and related bioactive properties of natural products, including structure–bioactivity correlations, mechanisms of action, the search of new sources of antioxidants, and the impact of botanical, geographical, and processing factors on the levels of these compounds.

A remarkable amount of work addressing distinct structural features capable of modulating the antioxidant properties of natural compounds through redox and non-redox-related pathways was accomplished by a group of researchers from the Guangzhou University of Chinese Medicine (Guangzhou, China). One of these studies was carried out with moracin C and iso-morain C (in which the distinct bond position causes different degrees of conjugation on the 2-phenyl benzofuran structure), leading to the conclusion that the presence of a double bond at the conjugation position increases the electron-transfer and H^+^-transfer potential [1]. Similarly, Li et al. demonstrated that the presence of an exocyclic double bond at the π–π conjugative site of oligostilbenes is a determinant feature to ensure good antioxidant capacity mediated by redox mechanisms [2]. In addition, the effect of geometric configuration on the bioactive properties of stilbenes was reinforced by Lin and coworkers, who showed that the preferential non-planar conformation of *Z*-resveratrol (with two dihedral angles blocking the extension of the conjugative system) hampered antioxidant redox-related pathways and consequently decreased the protection towards oxidative damage of bone marrow-derived mesenchymal stem cells, relative to *E*-resveratrol [3]. Notably, the evaluation of the two isomeric phenolic polyamines kukoamine A and B in such chemical and cellular models also elucidated the relevance of the positional isomeric effects on the biological properties of the two kukoamines [4]. 

Attention was given to the impact of substituents on the modulation of the bioactivity of phenolic compounds. In this context, the estimation of the antioxidant ability of 16 natural xanthones in the 2-phenyl-4,4,5,5-tetramethylimidazoline-1-oxyl 3-oxide radical trapping model revealed that this activity, which in these compounds may be mediated by electron-transfer and H^+^-transfer mechanisms, is mostly dominated by the substituent types *para*-di-OHs, 5,6-di-OHs, 6,7-di-OHs and 7,8-di-OHs [5]. Moreover, the comparison of the antioxidant properties of acteoside, forsythoside B, and poliumoside emphasized the importance of sugar residues in modulating the phenolic antioxidant capacity of phenylpropanoids, concluding that, although the apiosyl or rhamnosyl moieties might hinder electron-transfer and H^+^-transfer pathways, they overall improve the antioxidant and cytoprotective effects [6].

Another research direction exemplified in this Special Issue was intended to open new insights on the mechanisms of protection of natural compounds. In this regard, a theoretical study focused on Vam3 (a natural product similar to resveratrol) provided evidence that this compound can hinder lipid oxidation through ·OOH free radical scavenging even more effectively than resveratrol [7]. In addition, the work of Yang et al. elucidated that the mechanism underlying the protective effect of sulforaphane towards oxidative damage and apoptosis in cadmium-induced mouse Sertoli cells testis occurred through the activation of the Nrf2/ARE signal transduction pathway [8]. Also, proanthocyanidins were shown to protect the epithelial cells of the small intestine in mice from oxidative stress and apoptotic events caused by the mycotoxin zearalenone, via the inhibition of the endoplasmic reticulum stress-induced apoptosis pathway [9].

Phytochemical studies supporting the medicinal use of diverse natural products and their exploitation as novel sources of health-promoting compounds have been accomplished by several authors. Notably, most of them focused on phenolic compounds, which were described as good antioxidant agents or able to exert diverse biological properties, including cytoprotection, hepatoprotection, DNA damage protection, and modulation of key enzymes with impact in diabetes and obesity. Natural sources exploited in the presented studies comprise tubers, (flesh, peel, and whole tubers parts of yacon [10] and the orange fleshed sweet potato [11]) along with red corn cobs [12] and caryopses, flour, and leaves of colored rice (*Oryza sativa* L.) [13], several medicinal plants (*Tetraglochin ameghinoi* (Speg.) Speg. [14], *Flemingia philippinensis* Merr. et Rolfe [15], *Scabiosa stellate* L. [16], *Rhodiola rosea* L. [17], *Salvia elegans* Vahl., *Salvia greggii* A. Gray, *Salvia officinalis* L. [18], and *Retama raetam* (Forssk.) Webb [19]), two industrial by-products (grape pomace [20] and mango peels [21]), bee pollen [22], and humic acids isolated from peat [23]. Notably, Saada et al. underlined the variation of *R. raetam* (Forssk.) Webb bioactive components as a function of the plant cycle, concluding that the highest levels of polyunsaturated fatty acids, vitamin C, and phenolic compounds are present in its vegetative stage [19]. Moreover, Kim and coworkers contributed to the knowledge of protein pattern modifications during wheat germination and their relationship to the antioxidant properties. Longer germination times resulted in wheat samples with improved antioxidant capacity, which was closely related to the contents of phenolic acids, GABA, and proteins, such as glutathione S-transferase, granule bound starch synthase, and β-glucosidase [24].

The effects of preservation treatments and storage on the levels of bioactive compounds and the antioxidant properties of food products were also addressed by some contributing authors. When evaluating the effect of microwave vacuum drying (2.0 kW, 1.5 kW, and 1.0 kW) on the drying characteristics and quality attributes of green coffee beans, Dong et al. concluded that thermal processing increased the total phenolic content and antioxidant activity of the beans, particularly of those treated at higher microwave power [25]. In turn, Rafal et al. demonstrated that the hydrothermal sterilization of canned white beans caused various changes in their contents of nitrogen substances and partial loss of phenolic compounds and of antioxidative activity, although storage of the sterilized product could restore part of the antiradical activity of the seeds [26]. On the other hand, the study of Vieira et al., performed with thermal pasteurized and high-pressure processed juices over a 36 day storage period, highlighted the advantages of high-pressure processing for orange juice preservation, also allowing the enhancement of their nutritional benefits by increasing the content of some bioactive components [27]. 

Finally, the exploitation of the antioxidant properties of natural products aimed at the production of functional products was also approached. Zimniewska et et. proved that flax and hemp fibers have antioxidant properties inherent to the plant variety, fiber extraction method, and subsequent steps in the technological chain applied to fiber processing, which are elements to be considered in the design of functional tissues capable of supporting the protection of human skin against reactive oxygen species and UV radiation [28]. In another study, Adamenko et al. evaluated the possibility of the use of Cornelian cherry juice for the manufacturing of fruit meads with health benefits, concluding that among three cultivars differing in fruit color, namely, “Yantaryi” with yellow fruits, “Koralovyi” with coral fruits, and “Podolski” with red fruits, the latter showed significantly increased levels of active iridoids and phenolic acids as well enhanced antioxidant properties of the final product [29].
